# Metastasis of osteosarcoma to the trapezius muscle: a case report

**DOI:** 10.1186/1477-7819-12-176

**Published:** 2014-06-04

**Authors:** Yuichiro Sakamoto, Masahiro Yokouchi, Satoshi Nagano, Hirofumi Shimada, Shunsuke Nakamura, Takao Setoguchi, Ichiro Kawamura, Yasuhiro Ishidou, Akihide Tanimoto, Setsuro Komiya

**Affiliations:** 1 Department of Orthopedic Surgery, Graduate School of Medical and Dental Sciences, Kagoshima University, 8-35-1 Sakuragaoka, Kagoshima 890-8520, Japan; 2 The Near-Future Locomotor Organ Medicine Creation Course, Graduate School of Medical and Dental Sciences, Kagoshima University, 8-35-1 Sakuragaoka, Kagoshima 890-8520, Japan; 3 Department of Medical Joint Materials, Graduate School of Medical and Dental Sciences, Kagoshima University, 8-35-1 Sakuragaoka, Kagoshima 890-8520, Japan; 4 Department of Molecular and Cellular Pathology, Kagoshima Graduate School of Medical and Dental Sciences, Kagoshima University, 8-35-1 Sakuragaoka, Kagoshima 890-8520, Japan

**Keywords:** Osteosarcoma, Skeletal muscle metastasis, Resection, Chemotherapy

## Abstract

Metastasis of a primary osteosarcoma to the muscles is extremely rare. As there have been few reported cases, the necessity of surgical treatment for such metastatic lesions remains controversial. We present the case of a primary osteosarcoma with development of a solitary metastasis to the trapezius muscle during chemotherapy for pulmonary metastasis. The patient was a 51-year-old man diagnosed with osteosarcoma of the right tibia. After undergoing chemotherapy and femoral amputation, he developed pulmonary metastasis. Chemotherapy was reinitiated, however, after approximately 1 year a palpable tumor was identified in the patient’s right shoulder. This tumor grew and was associated with pain in the right shoulder. It was surgically removed 3 years after the re-initiation of chemotherapy. The pathological diagnosis was osteosarcoma with metastasis to the trapezius muscle. Although the patient died of respiratory failure due to pulmonary metastasis 14 months after resection of the metastatic lesion in the trapezius muscle, no new extrapulmonary metastasis was observed after the resection.

## Background

The metastasis of osteosarcoma is mainly hematogenous and it has been demonstrated that, at the time of diagnosis, approximately 80% of osteosarcomas have already metastasized if micrometastases are included [1]. The vast majority of metastases occur in the lungs, with metastasis to the soft tissue being extremely rare. We were only able to identify nine reported cases of metastasis to skeletal muscle in our review of the English literature. Here, we present a case in which the patient developed metastasis to the trapezius muscle while undergoing treatment for osteosarcoma.

## Case presentation

**Figure 1:**
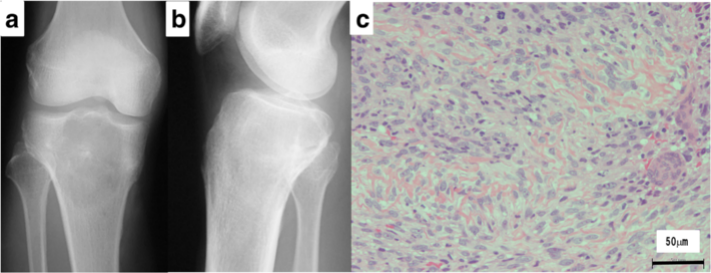
**Plain radiography and histological findings of primary osteosarcoma. (a)** Anteroposterior and **(b)** lateral radiographs show a central osteolytic lesion with partial ossification in the region from the proximal tibial epiphysis to the metaphysis. **(c)** Microscopic examination of the biopsy specimen revealed atypical spindle-shaped tumor cells and multi-nucleated osteoclast-like giant cells associated with a neoplastic osteoid formation in the interstitial tissue.

The patient, a 51-year-old man, consulted a local hospital after noticing idiopathic pain in his lower right thigh. The patient had no medical history of note. A radiography revealed osteolysis with partial ossification (Figure 1a and b) in the region from the proximal tibial epiphysis to the metaphysis. The patient was referred to our department where a magnetic resonance imaging (MRI) scan revealed a bone tumor located in the proximal tibia. A tissue biopsy was performed, revealing atypical spindle-shaped tumor cells and multi-nucleated giant cells, as well as a neoplastic osteoid formation in the interstitial tissue, which led to the diagnosis of osteosarcoma (Figure 1c).

**Figure 2:**
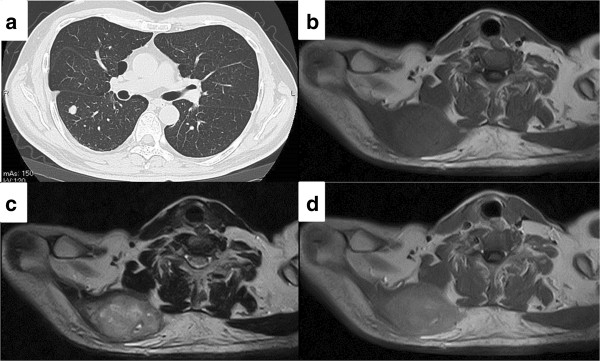
**CT and MRI imaging of metastatic osteosarcoma. (a)** Computational tomography (CT) imaging of lung metastasis in the early course of the disease. Multiple metastases are seen in both lungs. Axial **(b)** T1-weighted and **(c)** T2-weighted magnetic resonance imaging (MRI) scan shows a heterogeneous, ill-defined mass measuring 73 × 45 × 60 mm in the right trapezius muscle. **(d)** Gadolinium-enhanced T1-weighted image shows a circumscribed signal abnormality in the right trapezius muscle that was moderately enhanced.

**Figure 3:**
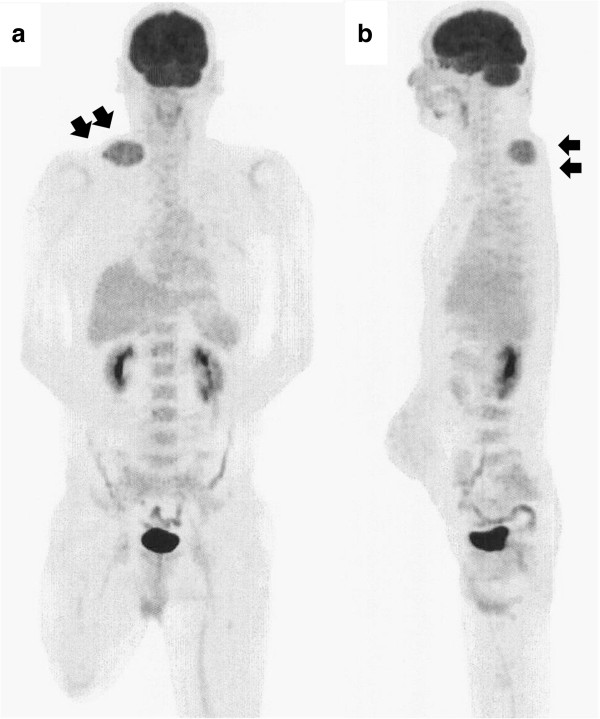
**FDG uptake in the trapezius muscle but not in the pulmonary metastasis. (a)** Anteroposterior and **(b)** lateral maximum intensity projection PET image demonstrates slight heterogeneous FDG uptake in the tumor located in the trapezius muscle (SUV = 4.6). Accumulation of the tracer in the pulmonary metastases was insignificant, and accumulation suggestive of metastasis was not found on the PET-CT scan, with the exception of the trapezius muscle.

**Figure 4:**
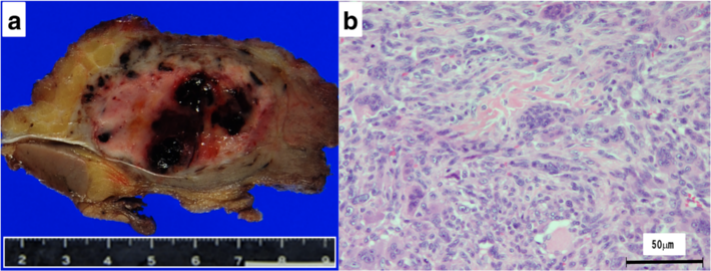
**Macroscopic and histological findings of the excised specimen. (a)** Gross appearance of the resected tissue showing a solid, white-yellowish tumor with bleeding and necrosis. **(b)** Microscopic examination of the resected specimen revealed marked proliferation of atypical spindle-shaped tumor cells with neoplastic osteoid formation. As with the biopsy specimen and the total cleavage H & E specimen, metastatic osteosarcoma was diagnosed. H & E, hematoxylin and eosin.

**Figure 5:**
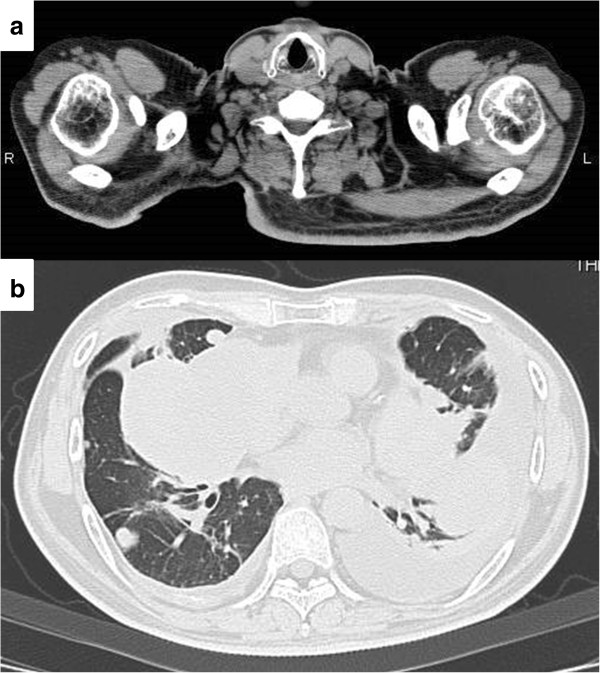
**CT imaging 14 months after resection of the muscle metastasis. (a)** No local recurrence was observed. **(b)** The pulmonary metastases began to grow rapidly.

At this time no apparent metastatic lesions were observed. The administration of preoperative neoadjuvant chemotherapy with cisplatin (120 mg/m^2^ on day 1) and doxorubicin (30 mg/m^2^ on days 1 to 2) was not effective and the tumor continued to grow. On completion of two courses of chemotherapy a pathological fracture occurred and an amputation of the right femur was performed. The histological response rate of the resected specimen was less than 10% of the entire lesion. Following surgery, five courses of adjuvant chemotherapy with ifosfamide (3 g/m^2^ on days 1 to 4) and VP-16 (75 mg/m^2^ on days 1 to 4) were administered and the patient was discharged as disease free.During follow-up, a computed tomography (CT) scan at 9 months after discharge revealed multiple metastases in both lungs (Figure 2a), and chemotherapy was re-initiated. The chemotherapy did not result in any marked change in the pulmonary metastases. Although the patient’s general condition continued to be good, a tumor was palpated in the right shoulder approximately 1 year later. At approximately 3 years after recommencing chemotherapy, the tumor had grown to 7 cm in size and was causing intense pain. An MRI scan revealed a well-defined and markedly enhanced mass located in the trapezius muscle (Figure 2b, c, and d). Although positron emission tomography-CT (PET-CT) scan revealed an insignificant accumulation of the tracer in the pulmonary metastatic lesions (Figure 3), the accumulation was comparatively high (standardized uptake value (SUV) of 4.6) in the tumor within the trapezius muscle (Figure 3). With the exception of the trapezius muscle, any accumulation suggestive of metastasis was not found on the PET-CT scan. A thallium-201-scintigraphy revealed a high and even accumulation of the tracer within the tumor in the trapezius muscle. A thermography revealed a 1.5 degree increase in temperature inside the tumor compared with the contralateral side, which was suggestive of high activity.Based on these results, the tumor within the trapezius muscle was deemed to be malignant. As no growth of the pulmonary metastases or development of new lesions was observed, the tumor in the trapezius muscle was surgically removed. An intraoperative review of the tissue specimen by a pathologist showed atypical cell proliferation and osteoid formation, which led to a diagnosis of metastatic osteosarcoma. Consequently, wide and tumor-free resection was performed. Macroscopically, the resected tissue consisted of a solid tumor with bleeding and necrosis (Figure 4a). As with the biopsy specimen and the total cleavage hematoxylin and eosin stain (H & E) specimen, metastatic osteosarcoma was diagnosed (Figure 4b).The integrity of other tissues around the surgery area was damaged (Figure 5a) and the range of motion of the patient’s shoulder joint was limited after surgery (flexion: 120°, abduction: 105°) compared with the non-operated shoulder joint. However, the patient’s pain was eased after resection of the trapezius muscle metastatic lesion, and a good quality of life was maintained between chemotherapy treatments. Despite chemotherapy, at 14 months post-resection the pulmonary metastases began to grow rapidly (Figure 5b), and the patient died from respiratory failure. No local recurrence (Figure 5a) or new extrapulmonary metastases were observed after resection of the metastatic lesion in the trapezius muscle.

## Discussion

It has been demonstrated that the metastasis of malignant tumors to skeletal muscle is extremely rare, with an incidence of approximately 1% [2,3]. According to Sridhar *et al.*[4], muscular metastasis is rare because of the following: (1) the high permeability of tumor cells and great variation in blood flow make it difficult for tumor cells to implant; (2) the movement of skeletal muscles physically destroys tumor cells; and (3) lactate metabolism within muscles and pH-dependent protease activity inhibit tumor cell proliferation in this type of tissue. Lung cancer is the most common primary malignancy to cause skeletal muscle metastasis, followed by hematological malignancies and gastrointestinal cancer [5,6], with muscular metastasis of bone and soft tissue tumors is extremely rare.

Previous reports have demonstrated that the incidence of soft tissue metastasis due to osteosarcoma is approximately 2% [7-11]. However, this figure includes cases with metastasis to the skin and subcutaneous fat; the actual rate of skeletal muscle metastasis is expected to be much lower. In our review of the English literature pertaining to osteosarcoma with skeletal muscle metastasis, only nine cases have been reported to date [7,12-18], mostly involving patients who were in a pre-terminal condition with multiple metastases. Contrastingly, in the present case, the pulmonary metastases were controlled and the patient had a good performance status. We believe that this represents an extremely rare case with solitary muscle metastasis localized to the trapezius muscle.

At present, conservative treatment with the systemic administration of anticancer agents along with radiation therapy is often chosen to treat metastatic lesions, and the necessity of surgical treatment remains controversial. Yamada *et al.*[12] reported a case of osteosarcoma with long-term survival after the resection of a solitary muscle metastasis and concluded that clinical decisions must be made on a case-by-case basis. In the present case, our patient continued to receive systemic anticancer agents after the appearance of a metastatic lesion in the trapezius muscle, but this lesion continued to grow despite good control of the pulmonary metastases. Because there have been few reported cases it is difficult to compare the validity of the metastatic lesion resection that we performed with radiation therapy. However, as a result of the resection the patient was relieved from severe pain, and did not experience any subsequent localized recurrence in the time leading up to his death from respiratory failure more than one year after the tumor resection. We believe that resection of the muscular metastasis in this patient was an effective treatment that enabled him to maintain a good quality of life.

Our patient received systemic chemotherapy for both pulmonary and muscle metastases. Despite achieving good control of the pulmonary metastases, we were unable to control the metastatic lesion in the muscle with chemotherapy. The resected muscle lesion revealed living cells in the majority of the tissue. A previous study demonstrated differing sensitivity patterns to cytostatic agents in cell culture studies of metastatic pulmonary and muscular osteosarcoma, suggesting that therapy may have resulted in positive selection for a tumor clone which is resistant to therapy and/or more metastatic than other subpopulations within the tumor [17]. Although treatment experience is limited owing to the rarity of this condition, our case report illustrates that, depending on the patient’s overall condition, resection might be considered as a therapeutic option when treating osteosarcoma with muscle metastasis that does not respond to chemotherapy.

## Conclusions

We have presented an extremely rare case of primary osteosarcoma with solitary metastasis to the trapezius muscle that developed during chemotherapy for pulmonary metastasis.

## Consent

Written informed consent was obtained from the patient for publication of this case report and any accompanying images. A copy of the written consent is available for review by the Editor-in-Chief of this journal.

## Abbreviations

CT: Computational tomography; H & E: Hematoxylin and eosin stain; MRI: Magnetic resonance imaging; PET-CT: Positron emission tomography-CT; SUV: Standardized uptake value.

## Competing interests

The authors declare that they have no competing interests.

## Authors’ contributions

SY, SN, HS, and MY managed the case and drafted the manuscript. SN, TS, IK, and YI collected the findings and participated in the design of the study. AT performed pathological examination and figure preparation. SK was involved in the design and coordination of the study and helped to draft the manuscript. All authors have read and approved the final manuscript.
